# Veterinary Department—University of Pennsylvania

**Published:** 1887-07

**Authors:** 


### VETERINARY DEPARTMENT—UNIVERSITY OF PENNSYLVANIA.

We had always been proud of the Commonwealth of Pennsylvania as a great agricultural State, for we knew that the agricultural industries of the Commonwealth paid four-fifths of the taxes, and that the Granger vote was an important element in State politics ; but our pride has had a fall. We had already commenced an editorial to notify our readers that the Legislature of Pennsylvania had passed an appropriation of fifty thousand dollars for the construction of new buildings on the grounds of the Veterinary Department of the University of Pennsylvania. We expected to advise the student from away, that dormitories would await his coming. We planned that cattle stables should furnish opportunity for practical instruction in obstetrics and zootechnics. We were ready to announce a farriery, where blacksmiths could come and in a three months' course learn that one subject so essential, but almost unknown to them, the principles of the horse's foot. Eight free scholarships were to have a perpetual foundation. All these could have been supplied to the public by the fifty thousand dollars which the representatives of a great agricultural state willingly voted for, urged to do so by their constituents, and only by an accident failed to make it one hundred thousand. The latter sum would have completed the institution with a botanical garden, where the veterinary student could learn to recognize the crops that grow on his patron's farm, the indigenous remedies and noxious weeds, which are found wherever the practitioner is called to see a herd of cattle or a flock of sheep.

However, His Excellency, the Governor of the Commonwealth, found a Hahnemann School and a Mushroom Medical School in Philadelphia, which has not a dollar invested, more needful to the public good, and placed his veto on appropriations to the Veterinary School, the State Agricultural Society and for the meeting place of the State Grangers. The failure to obtain the means for the necessary additions to the Veterinary School, occurred at a critical moment and will prove a serious inconvenience, but only a temporary one, as the entire people of Pennsylvania showed great interest in this undertaking, and when once they appreciated the scope which veterinary medicine is capable of, realized the absolute necessity it is to the country.

We can, however, announce with pleasure the graduation of the first class. Ten gentlemen received at the commencement of the University of Pennsylvania, on June 8th, their diplomas—"Veterinariæ Medicinæ Doctor." At the opening of the Veterinary Department three years ago, thirty-three matriculants were found on the roll. During the first year sixteen found the studies more than they anticipated, and failed to pass their examinations; the second year's examinations reduced the number to thirteen, and ten successful candidates were the result of the final examinations, after twenty-seven months' study. The fine physical appearance of the new veterinarians was the subject of much comment and applause as they walked on the stage of the Academy of Music among the two hundred students of the other departments, who were also receiving the reward of their respective studies. This commencement marks an era in the advance which veterinary medicine has been making in the United States for the past ten years. The public reception of these men, side by side with the graduates of other scientific branches of study, will do much to break down the last vestige of the vulgar prejudice which has existed in regard to the veterinarian.

H.

---

An Explanation.—The *Veterinary Journal* for March contains a communication from "Amicus" in which Dr. Burke, of India, is bitterly attacked for having expressed his high appreciation of the *Journal of Comparative Medicine and Surgery* in a letter to the editors of that periodical.

"Amicus" also accuses us of having published an extract from this letter as an advertisement. The accusation is entirely false, the extract in letter form, having been sent only to a few friends of Dr. Burke's in England.

The May number of the *Veterinary Journal* publishes a reply from Dr. Burke in which he says: "Without attempting to discuss the reasons for my opinion regarding the merits and general finish of the journal in question, as it is now issued by its present editors, I may only refer to your own (the editor's) remarks in the journal for March, which should be a sufficient guarantee in itself to secure a not too narrow circle of readers for the *Journal of Comparative Medicine and Surgery* wherever the English language is spoken.

"I may inform 'Amicus' that, were he to publish a journal of the same scientific tone and high standard as the one referred to, I would gladly subscribe for it as for any other of equal merit."

The editor's remarks on this journal referred to above, are as follows :

"In October last, what might be designated a new series of this quarterly periodical was begun, and the alterations which have been made in its arrangement and production will render it yet more acceptable to the wide class of readers it commands as a representative scientific journal of its class." C.

Hydatids in a Camel.—In the winter of 1883, while Demonstrator of Equine Anatomy at the Columbia Veterinary College of New York, I had occasion to examine the viscera of a camel that had died at the Central Park Zoological Garden. In the lungs, liver and mesenteries were a number of spherical masses which proved on further examination to be hydatid cysts. The largest had a diameter of 3 c. m; ; others were nearly as large. The identification of the cystic contents was made by Dr. Satterthwaite of the New York Post Graduate Medical College.

These hydatids can in no way be accounted as indigenous to this country, for the cysts are of slow growth and the camel may have been infected before arriving on our shores.

Cooper Curtice, D. V. M.

